# Neuropsychological predictors of vocational rehabilitation outcomes in individuals with major depression: A scoping review

**DOI:** 10.3389/fpsyt.2022.942161

**Published:** 2022-11-09

**Authors:** Juliane Bergdolt, Pauline Sellin, Martin Driessen, Thomas Beblo, Lorenz B. Dehn

**Affiliations:** ^1^Department of Psychiatry and Psychotherapy, Evangelisches Klinikum Bethel, Universitätsklinikum OWL of Bielefeld University, Bielefeld, Germany; ^2^Department of Psychology, Bielefeld University, Bielefeld, Germany

**Keywords:** supported employment (SE), cognitive function, depression, work, neuropsychology

## Abstract

**Background:**

Major depression is one of the leading causes of disability and limited capacity to work. Neuropsychological impairment is a common symptom in acute and remitted major depression and is associated with poor psychosocial functioning. This scoping review aimed to identify research on the role of neuropsychological functioning in outcomes of vocational rehabilitation programs in individuals with depression.

**Methods:**

We report on the conduct of this pre-registered (https://osf.io/5yrnf) scoping review in accordance with PRISMA-ScR guidelines. PubMed and PsychInfo were systematically searched for English or German research articles published between 1990 and September 2021 that studied objective neuropsychological tests as predictors of vocational rehabilitation interventions and included participants with depression.

**Results:**

The systematic literature search yielded no studies that specifically targeted subjects with major depression. However, eight articles published since 2016 were included in the review, analyzing data from five trials that evaluated the effectiveness of supported employment in North America and Europe in severe mental illnesses. An estimated 31% of the total number of participants included (*n* = 3,533) had major depression. Using a variety of cognitive tests and covariates, seven articles found that neuropsychological functioning – especially global cognition scores, verbal and visual learning and memory – significantly predicted vocational outcomes of rehabilitation programs.

**Conclusion:**

Despite a lack of studies specifically targeting major depressive disorder, the identified literature suggests that higher baseline neuropsychological functioning predicts better vocational outcomes of supported employment programs in individuals with depression. In clinical practice, additional neuropsychological modules during return-to-work interventions might be helpful for vocational outcomes of such programs.

## Introduction

Major depressive disorder is on top of the world’s most prevalent mental health problems and is associated with poor health, mortality, disease and increased disability (1, 2). Major Depression, even in remission, also affects working life and is related to unemployment, reduced work productivity, absenteeism and presenteeism (3), accounting for a high economic burden across different countries (4, 5). Moreover, depression is related to an increased risk for early retirement and applying for disability pension (6, 7).

The major symptoms of depression are depressed mood and loss of interest or lack of pleasure, but it also comes along with several cognitive impairments like diminished ability to think, reduced concentration and attention, or indecisiveness (8, 9). Subjectively perceived cognitive deficits have been associated with perceived global disability, workplace performance, and quality of life (10). Meta-analysis have confirmed objective neuropsychological deficits in depressive disorders in the domains of attention, memory and executive function (11). There is evidence that cognitive dysfunction across several neuropsychological domains is already identifiable in the first episode of depression (12). In addition, cognitive impairment was shown to persist even in remitted patients (12, 13) and appeared to be a long lasting residual-symptom (14) that increases the risk of relapse (15).

Neuropsychological deficits are negatively related to various aspects of psychosocial functioning, treatment progress, quality of life and occupational outcomes (16–19). Recent reviews suggest that neuropsychological deficits may be a principal predictor of occupational functioning (17, 20, 21). Specifically, one review found that employment status and work impairment were associated with neuropsychological deficits across several domains, e.g., attention, executive functions and verbal memory in two depressed samples (17). Clark et al. (20) concluded that cognitive dysfunction in various domains may have a direct impact on work productivity, especially on presenteeism in depressed people. Woo et al. (21) found evidence that residual deficits in memory, attention, learning and executive function predict occupational impairment even in people with remitted depression.

To support people with major depression returning to work or reducing work disability (e.g., sickness absence), several rehabilitation approaches have been studied (22). A recent review (22) about vocational rehabilitation interventions for people with depression showed overall possible positive effects of a combination of work-directed and psychological interventions in reducing illness-related absence days compared to care as usual, but no difference was found with regard to the amount of people who worked at follow-up. Given the emerging evidence about the association between cognitive functioning and vocational functioning in acute and remitted major depression, neuropsychological variables may also affect outcomes of vocational rehabilitation interventions. Evidence comes from studies of people with psychotic disorders and supported employment (SE) programs, like the evidence-based Individual Placement and Support model (IPS) that provides rapid job search based on individual preferences and skills, and long-term support at the workplace (23). IPS and other programs using SE like the Program for Assertive Community Treatment (PACT) have been shown to significantly improve vocational outcomes for people with severe mental illnesses (24, 25). Research in schizophrenia suggests that more severe cognitive impairment predicts worse work and SE outcomes, including the number of hours worked, job tenure and wage levels (26–32). For example, higher scores in the subdomains of executive functions (32), visual organization and memory (26), and verbal learning and memory (28) have been identified to significantly predict better rehabilitation outcomes in addition to general cognitive function. Accordingly, additional cognitive training has been examined in people enrolled in SE (33). In samples with psychotic or bipolar disorders receiving SE, there is evidence for the effectiveness of additional cognitive training for gaining competitive employment and other work-related outcomes, further supporting the hypothesis that cognitive functioning influences the success of vocational rehabilitation interventions (34–39). For instance, over 2–3 years, patients in the SE with cognitive training program were more likely to work, held more jobs, worked more weeks and hours, and earned higher wages than patients who received SE alone (38).

In sum, neuropsychological impairment is a common and meaningful symptom in major depressive episodes, and SE interventions that help people with SMI to return to work have been shown to be effective. In addition, a relationship between neuropsychological performance and work outcomes has already been demonstrated for some psychiatric and neurological patient samples [e.g., (40–42)]. However, it is unclear whether and how these known neuropsychological impairments might specifically affect the benefits of return-to-work interventions for individuals with major depression. Addressing these issues might have important healthcare implications, given the high prevalence of major depression and the occupational limitations associated with this disorder. It is unclear if the association between neuropsychology and rehabilitation outcomes also holds for SE participants with psychiatric diagnoses other than psychosis, because persons with schizophrenia show more severe cognitive deficits than for example severely depressive patients (43). Learning more about the relationship between neuropsychological variables and vocational rehabilitation program outcomes in people with major depressive disorder is crucial for the development and adjustment of occupation-related interventions, such as SE, for people with depression. If neuropsychological functioning could be shown to affect outcomes of vocational rehabilitation programs, diagnostic assessments and interventions targeting cognition can be used to enhance work-related outcomes and improve the quality of life of people with major depressive disorder. Following a scoping review approach, we aimed to identify existing research on the association between cognitive functioning and outcomes of vocational rehabilitation interventions in people with depressive disorders. We conducted a scoping review to provide an overview of the scope and nature of research on neuropsychological variables as predictors of the success of return-to-work interventions for people with major depression. Scoping reviews aim to identify and map the existing research on a particular topic that is usually broadly defined. Following a systematic literature search they may identify research gaps, key concepts, theories, or sources of evidence (44). Specifically, we aimed to answer the following research questions:

• What research is available regarding the association between neuropsychological functioning and vocational rehabilitation intervention outcomes in people with depressive disorders, what were study contexts and which main research questions were posed?
• Which rehabilitation interventions have been examined?
• Which neuropsychological domains and tests have been investigated?
• What vocational outcomes have been used and which major results have been found?

## Methods

### Protocol and registration

The study protocol was prospectively registered with the Open Science Framework on September 9, 2021.^1^ This scoping review was performed using the Joanna Briggs Institute guidance for the conduct of scoping reviews (44). We report the conduct and results of this scoping review in accordance with the Preferred Reporting Items for Systematic Reviews and Meta-Analyses (PRISMA) extension for scoping reviews (45).

### Eligibility criteria

Original research studies published between 1990 and 2021 in English or German language were included that (a) had a quantitative-empirical study design, (b) had examined persons with a depressive disorder and, if applicable, a comparison or healthy control group, (c) examined a vocational rehabilitation intervention, (d) measured, among other characteristics, neuropsychological variables prior to the intervention and (e) statistically correlated outcome criteria with baseline variables following the intervention. Reviews published between 1990 and 2021 in English or German language were included if they examined the association between neuropsychological variables and outcomes of vocational rehabilitation interventions in people with major depressive disorder. Studies were excluded, if they did not include patients with depressive disorders, did not conduct an occupational or work-related intervention, or did not provide information on baseline neuropsychological variables and/or vocational outcome parameters.

### Search strategy and study selection

PubMed and PsychInfo were searched for relevant articles using combinations of search terms for the three relevant topics neuropsychological performance AND work-related rehabilitation AND depressive disorders. Specifically, for neuropsychological performance the following search terms were used: [(“cognition”) OR (“cognitive function*”) OR (“cognitive impairment”) OR (“cognitive dysfunction”) OR (“cognitive deficit”) OR (“neurocog*”) OR (“neuropsycholog*”) OR (“memory”) OR (“executive function*”) OR (“verbal learning”) OR (“processing speed”)].

Regarding work-related rehabilitation interventions, the following terms were used: [(“supported employment”) OR (“individual placement and support”) OR (“individual enabling and support”) OR (“vocational rehabilitation”) OR (“work-focused”) OR (“job acquisition”) OR (“job coaching”) OR (“occupational rehabilitation”) OR (“work-related”) OR (“vocational rehabilitation”) OR (“work-directed”) OR (“vocational training”) OR (“prevocational training”) OR (“vocational intervention”)].

Finally, search terms for depressive disorder were: [(“major depression”) OR (“depression”) OR (“depressive disorder”) OR (“major depressive disorder”) OR (“depress*”)].

To find studies that include all three topic areas, AND operators were used between each combination of terms for the topics. The search query was adapted for the respective requirements of each database. For the PubMed search query, terms were searched in [All Fields]. The initial search was conducted on September 10, 2021. In addition, reference lists of the included manuscripts were searched for further relevant studies. During citation searching, we noticed a couple of studies examining neuropsychological variables among several other predictors of vocational rehabilitation outcomes. As scoping reviews may follow an iterative search strategy (44) and to avoid overlooking neuropsychological predictors in this study area, we performed a further PubMed search, using the following search terms: (“predict*”[Title/Abstract]) AND (“IPS”[Title/Abstract] OR “individual placement and support”[Title/Abstract] OR “vocational rehabilitation”[Title/Abstract] OR “supported employment”[Title/Abstract]) AND (“mental illness*” [Title/Abstract] OR “depressive disorder”[Title/Abstract]).

First, two authors (LD and JB) independently screened titles and abstracts of the literature search for eligibility. Full texts of articles that had not been excluded in the first step, were independently reviewed by LD and JB with respect to the research question. Ambiguities were discussed and resolved by joint consensus.

### Data charting and data items

Two authors (LD and JB) jointly developed a data charting form that was updated in an iterative process. Data were charted independently and results were discussed. First, variables to describe the study in terms of year of publication, country, study design, and sample characteristics were extracted. With regard to our research question, the following data were abstracted: main research question, neuropsychological domains and tests, other examined predictors, information about the studied vocational intervention, any occupational outcomes, main findings and findings regarding neuropsychological functioning predicting vocational rehabilitation intervention outcomes.

### Synthesis of results

First, we grouped basic information of the included articles in terms of authors, publication year, country, sample size, target group, and intervention. In a separate table, we mapped data regarding neuropsychological predictors, with neuropsychological tests used, main research questions, included covariates, follow-up period, and vocational outcomes variables. The results presented in the tables were then summarized narratively. To display the amount of participants with major depression included in the samples, we summarized the total number and percentages of diagnostic groups included in each articles’ sample. If articles did not report on exact diagnostic groups, we aimed to collect information about their sample in other published research papers about the trials. When subsamples were used and information on the exact number of participants with a diagnosis was not available for a specific subsample, we estimated the number of participants with a particular diagnosis by extrapolating the percentage of individuals with that diagnosis in the base sample.

## Results

**FIGURE 1:**
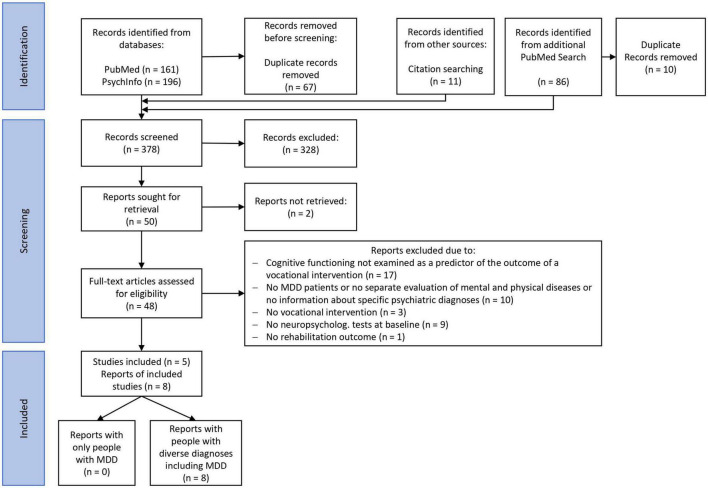
Preferred reporting items for systematic reviews and meta-analyses flow-chart.

The systematic literature search revealed no studies investigating neuropsychological predictors of vocational rehabilitation interventions specifically for persons with major depression. However, we identified eight articles related to our research question that examined diagnostically mixed samples including major depressive disorder. Due to the exploratory approach of scoping studies, these articles were still included if they examined individuals with depression in addition to participants with other psychiatric diagnoses. Figure 1 shows the search and inclusion process according to the PRISMA flow chart. The eight included reports analyzed data from five studies. Because the articles nevertheless focused on different aspects regarding neuropsychological predictors of work outcomes, we decided to consider all articles separately, even when they were based on the same sample.

All eight reports were original research articles published in peer-reviewed psychiatric and mental health (46–51), or neuropsychological (52) journals, including one brief report (47). Table 1 describes the included research articles in terms of publication year, country in which the sample was recruited, total analyzed sample size, target group, and vocational intervention. Although research published since 1990 was eligible for inclusion, all included articles were published between 2016 and 2021. Data have been collected in the context of five studies (53–57) evaluating SE or IPS interventions in people with SMI who were currently unemployed and looking for a job. Three studies (54–56) were conducted in North America (*n* = 2 USA, *n* = 1 Canada), while two (53, 57) were conducted in European countries (*n* = 1 Switzerland, *n* = 1 Denmark). Of the five studies, one was a longitudinal observational study without a control group (56), and four were randomized controlled trials evaluating different IPS interventions. The studied interventions varied substantially in terms of additional support provided besides the work-focused SE or IPS programs.

**TABLE 1:** Characteristics of included articles (*n* = 8).

Authors	Year	Country	*N*	Target group	Intervention	Follow-up
Burton et al. (52)	2019	USA	153	SMI	IPS plus cognitive training vs. IPS “enhanced”	2 years
Mahmood et al. (48)	2019	USA	153			

Metcalfe et al. (51)	2018	USA	1,004	Social Security Disability beneficiaries with SMI	IPS plus “a comprehensive package of mental health services, full insurance coverage, and suspended disability reviews” (49) vs. TAU	2 years
Metcalfe, Drake et al. (50)	2017	USA	2,055			
McGurk et al. (49)	2018	USA	945			

Corbière et al. (46)	2017	Canada	489	SMI	SE (24 different programs)	6 months

Christensen et al. (58)	2021	Denmark	720	SMI	IPS plus cognitive remediation and social skills training vs. IPS vs. TAU	18 months

Landolt et al. (47)	2016	Switzerland	116	SMI	IPS with different time budgets	2 years

SMI, severe mental illness; IPS, individual placement and support; articles that rely on data from the same trial are grouped together.

**FIGURE 2:**
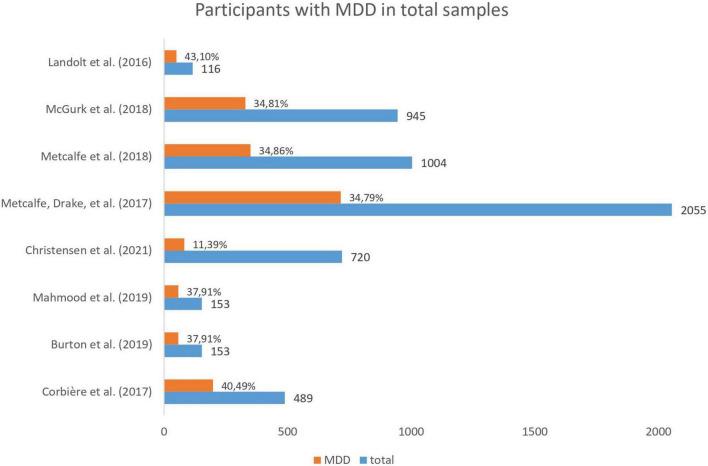
Total number of participants and percentage of people with major depressive disorder (MDD) included in each article.

According to the method sections of the articles, a total of *n* = 3,533 participants were included in the five studies. Sample sizes differed widely ranging from *n* = 116 (47) to *n* = 2,055 (50). Metcalfe et al. (51) and McGurk et al. (49) both analyzed sub-samples (intervention group) of a study with a total *n* = 2,055 that Metcalfe, Drake et al. (50) used for their analyses. All articles reported that the target group were people with SMI, but one study (54) only included people with SMI who received social security disability benefits. Figure 2 shows the total samples sizes and the percentage of people with major depression in each articles’ sample. Because Metcalfe, Drake et al. (50) and Metcalfe et al. (51) did not specify the amount of people with major depression, data from the same sample reported by McGurk et al. (49) were extrapolated to estimate the total number of participants with major depression and bipolar disorder in all studies (see 2.5 synthesis of results). According to this calculation, the total number of participants diagnosed with major depressive disorder across all five studies was approximately *n* = 1,103 (31%). The proportion of participants with major depressive disorder varied between 11% (58) and 43% (47). The most prevalent other primary diagnoses were psychotic disorders (total *n* = 1,373; 38%) and bipolar disorders (estimated total *n* = 852; 24%). The articles created diagnostic subgroups based on the primary diagnosis (e.g., major depressive disorder) or primary diagnostic group (e.g., affective disorders). As the articles did not report all their results separately for major depression and other diagnoses, the compiled findings on neuropsychological predictors of vocational interventions presented below include data from individuals with a primary diagnosis of major depression as well as from individuals with other primary SMI diagnoses. In one part of the results section, however, we summarize some analyses of differences between diagnostic groups on neuropsychological measures and vocational outcomes from individual studies.

Table 2 provides an overview of the main research questions, neuropsychological measures, vocational outcome variables, and covariates of the articles included.

**TABLE 2:** Information on neuropsychological predictors of work outcomes (*n* = 8).

Article	Main research question	Neuropsychological variables (as baseline predictors)	Vocational outcome variable	Multivariate covariates
***Research questions focusing on neuropsychological predictors of work outcomes***				
McGurk et al. (49)	“(…) we explored cognitive predictors of work in a diagnostically mixed sample of people with major mood and schizophrenia-spectrum disorders”	**Significant:** – Brief Assessment of Cognition in Schizophrenia (BACS) composite score – BACS verbal learning **Not significant (single scores):** – BACS verbal fluency – BACS digit sequencing – BACS token motor – BACS symbol coding – BACS tower of London	Average weekly hours worked	**Significant:** Work history, ethnicity **Not significant:** Diagnosis, gender, education, age
Mahmood et al. (48)	“(…) determining the strongest neuropsychological and other modifiable predictors of work outcomes in a large sample of IPS service users with varying diagnoses”	**Significant (greater weeks worked, if attained a job):** – Global Deficit Score (GDS) – Brief Visual Memory Test Revised (BVMT-R) **Significant (higher wages earned, if attained a job):** – Global Deficit Score (GDS) – Letters FAS verbal fluency – Brief Visual Memory Test Revised (BVMT-R) **Not significant:** – Trail Making Test (TMT) A and B – Wisconsin Card Sorting Test 64 (WCST-64) – Memory for Intentions Screening Test (MIST) – Continuous Performance Test (CPT) – Symbol coding and category fluency (BACS subscales) – Wechsler Memory Scale III Spatial Span (WMS-III SS) – University of Maryland Letter-Number Span (LNS) – Hopkins Verbal Learning Test Revised (HVLT-R) – Neuropsychological Assessment Battery (NAB) Mazes	– Job attainment – Weeks worked – Wages earned	**Significant (job attainment):** Work history Negative symptom severity **Significant (wages earned):** Work history **Not significant:** Positive symptom severity, education, racial/ethnic minority status, diagnosis, depressive symptom severity, functional capacity, intellectual functioning
Landolt et al. (47)	“The aim of the analysis was to test which cognitive parameters significantly affect entering and maintaining competitive employment in a sample of Swiss outpatients with mental illnesses who were receiving IPS”	**Significant:** – Verbal Learning and Memory Test (VLMT) learning – Factor II: VLMT Score – Factor III: S-words, DSCT, Digit Span, VLMT learning **Not significant:** – Verbal fluency test (S words and animals) – Digit Symbol Coding Test – Stroop Color-Word Interference Test – Digit Span from Wechsler-Adult-Intelligence-Scale (WAIS-III) – Factor I: Stroop-Tests	Competitive employment ≥ 1 day	Age, gender, duration of previous unemployment, Clinical Global Index, Global Assessment of Functioning scale *(no information about significance)*
Burton et al. (52)	“(…) we aimed to evaluate prospective memory performance in an employment-seeking sample of individuals with SMI and its relationship to real-world functional variables, including employment outcomes”	**Significant (weeks worked):** – Memory for Intentions Screening Test (MIST)	– Weeks worked – Wages earned	**Not significant:** Diagnosis, years of education, standard reading score
***Research questions focusing on various variables predicting work outcomes***				
Metcalfe et al. (50)	“(…) to identify client factors that predicted competitive employment and examine the potential mitigation effect of IPS supported employment on Social Security Disability Insurance beneficiaries with SMI”	**Significant (model without work history):** – Digit Symbol Coding Test (paper pencil)	Competitive job during follow-up	**Significant:** Hispanic, Health/SF-12, Work history, disability rolls **Not significant:** Ethnicity, gender, race, marriage, diagnosis, age, education, ER visits, quality of life
Metcalfe et al. (51)	“We determined the effects and relative importance of client characteristics, IPS fidelity, and local economic factors in a multivariable model of quarterly competitive employment among IPS recipients”	**Significant:** – Brief Assessment of Cognition for Schizophrenia composite score (BACS)	Competitive employment ≥ 1 day	**Significant:** Hispanic, work history, disability rolls, local unemployment rate **Not significant:** Gender, education, marriage, race, age, quality of life, health/SF-12, diagnosis, local GCP change, population density, local unionization rate
Christensen et al. (58)	“The aim of this study was to identify individual and sociodemographic factors that predict vocational recovery among people with severe mental illness in the Danish IPS trial and to investigate the potential advantages of participating in individual placement and support to overcome specific risk factors for unemployment”	**Not significant:** – Brief Assessment of Cognition in Schizophrenia (BACS) composite score	Obtain employment or education	**Significant:** Gender, age, work history, readiness for change **Not significant:** Social performance, Symptom severity, depression, self-efficacy, self-esteem
Corbière et al. (46)	“The main goal of this study was to identify the most salient employment specialist competencies and clients’ variables contributing to competitive employment for people enrolled in Canadian SE programs while controlling for the quality of the SE program implementation”	**Significant (model without employment specialist variables):** – Trail Making Test (TMT) A **Not significant:** – TMT B	Competitive job acquisition	**Significant:** Age, work history, job search strategies, working alliance **Not significant:** Gender, education, diagnosis, motivation, self-esteem, perceived barriers

Significance is indicated according to the multivariate models of the individual articles.

### Research questions and neuropsychological tests

With respect to the role of neuropsychological predictors in the research questions, two types of articles were identified. Main research questions of four articles (47–49, 52) explicitly focused on neuropsychological variables while the main research questions of the other four articles (46, 50, 51, 58) focused on various client or context variables predicting work outcomes of vocational rehabilitation programs, including neuropsychological predictors. In general, articles assessed a wide range of neuropsychological tests and cognitive domains. Articles used only one neuropsychological test (50) or various tests or test batteries (see Table 2). The most frequently used test (*n* = 4) was the Brief Assessment of Cognition in Schizophrenia (BACS), which contains the six measures list learning, digit sequencing task, token motor task, category instances and controlled word association tests, symbol coding test, and Tower of London (59). Two articles (51, 58) only analyzed the BACS composite score which is calculated across scores of all six measures, while one article (48) only used two subscale scores (symbol coding and category fluency) and one article evaluated both the composite and all six individual test scores (49).

### Results at bivariate level

All articles evaluated the role of cognitive functioning for predicting outcomes of vocational rehabilitation interventions in models with multiple predictor variables. Five articles (47–49, 51, 58) additionally reported bivariate associations between cognitive functioning and work outcomes. In all of them, some neuropsychological variables [BACS composite score, BACS-symbol coding, letter-number-span, Wisconsin card sorting test, digit span, and Verbal Learning and Memory Test (VLMT)] were significantly associated with work outcomes of the SE intervention. Additionally, Metcalfe, Drake et al. (50) reported that participants who were employed during follow-up performed significantly better on the baseline Digit Symbol Test than participants who were unemployed at follow-up.

### Results at multivariate level

Of eight articles included, seven found a significant predictive effect of any neuropsychological measure in multivariate models predicting work outcomes of the vocational rehabilitation program (46–52). Considering that some articles relied on the same samples, this implies that four out of five studies showed that higher baseline neuropsychological test scores predicted better outcomes of SE interventions. Individual test scores, as well as global cognition scores were analyzed.

#### Global cognition scores

Five articles used composite scores of individual neuropsychological tests to predict SE outcomes in models with multiple predictors, of which four observed significant results. Mahmood et al. (48) calculated a “global deficit score” (GDS) averaging cognitive impairment across all 13 individual measures and found that GDS significantly predicted average hours worked and total wages earned in participants who attained a job. Landolt et al. (47) conducted an exploratory factors analysis and found that the most general factor (digit symbol and digit span tests, verbal learning score, verbal fluency test) significantly predicted job attainment and maintaining a job. McGurk et al. (49) found that the BACS composite score significantly predicted average hours worked. Metcalfe et al. (51) and Christensen et al. (58) both evaluated if the BACS composite score predicted job attainment during the follow-up period, but only Metcalfe et al. (51) found it to be a significant predictor.

#### Individual tests

Among the individual tests assessed, mainly measures of verbal and visual learning and/or memory significantly predicted SE outcomes. In one article, the Brief Visual Memory Test Revised (BVMT-R) significantly predicted average weeks worked and wages earned (48). Furthermore, the VLMT utilized by Landolt et al. (47) was significantly associated with job attainment. Regarding the BACS subtests, McGurk et al. (49) showed that only verbal learning remained significant in a model including all predictors. Burton et al. (52) only studied one cognitive dimension, namely prospective memory measured by the Memory for Intentions Screening Test (MIST) and found it to be a significant predictor of weeks worked during 2 years of SE. However, the analysis of the same sample by Mahmood et al. (48) including many other predictors did not show a significant effect of the MIST score. Likewise, another verbal learning test (HVLT-R) (48) and short-term memory tests (digit span/spatial span) (47, 48) did not reach significance in other articles.

Besides verbal and visual learning and memory, verbal fluency was predictive of wages earned (48) and processing speed measured by the Digit Symbol Coding Test (50) or the Trail Making Test (TMT) A (46) was significantly associated with job attainment. However, when employment specialist variables were entered into the model, the TMT A score was not significant anymore in the article by Corbière et al. (46) and the Digit Symbol Coding Test only reached borderline significance when work history was entered into the model (50). At the bivariate level, the BACS-symbol-coding subtest has been significantly associated with the amount of work during follow-up (48, 49). However, in other articles, a digit symbol coding test (47) and verbal fluency tests (47, 49), were not found to be significant predictors of vocational outcomes of SE among other significant neuropsychological variables.

In general, the included articles suggest that tests measuring complex executive functions, like Tower of London (49), or Stroop test (47), were not among the significant single domains. Likewise, the TMT A and B which were intended to measure processing speed and executive functions, respectively, were not found to be significant predictors in another article (48).

### Covariates

The majority of articles included a measure of work history in their multivariate models (*n* = 7/8) (46–51, 58). The articles defined work history differently, with:

• four articles reporting a dichotomous measure, namely any paid work during the past 2 years (yes/no) (49–51), or at least 2 months of work within the last 5 years before baseline (yes/no) (58),
• and three articles reporting a continuous measure, namely time (months or years) of unemployment before study entry (46–48).

Work history significantly predicted work outcomes of SE at multivariate level in all these articles, with the restriction that Landolt et al. (47) did not report on the significance of covariates. Neuropsychological variables significantly added predictive power to models that also included work history in four of seven articles according to the alpha levels that the authors applied (47–49, 51). Metcalfe, Drake et al. (50) concluded that the Digit Symbol Coding test reached borderline significance when work history was included in the model.

With regard to demographic variables like age, gender, or ethnicity that many articles assessed, the results were inconclusive. No interaction effects with neuropsychological variables were investigated, except for diagnostic group.

#### Diagnostic group

In sum, all results reported were not exclusive for depressive participants and include data from mixed SMI samples with patients with major depressive disorder and other, mainly psychotic, diagnoses. This prevents us from reporting neuropsychological findings on SE outcomes as specifically related to people with major depression. However, some included articles analyzed diagnostic differences in their results, which are summarized here. Persons with psychotic disorders performed worse on some prospective memory subscales (52) and the BACS (49), and had a greater overall cognitive impairment score (48) than persons with affective disorders. However, diagnostic group (*n* = 3 affective disorders vs. psychotic disorders; *n* = 4 major depression vs. bipolar disorder vs. psychotic disorders) had no significant effect on SE outcomes in either article that entered it into the multivariate models (46, 48–52). Thus, some authors concluded that their results are valid independent of diagnosis (48, 49, 52). Only McGurk et al. (49) entered the interaction between neuropsychological predictors and diagnosis (affective disorder vs. psychotic disorder) into their predictive model. Because the interaction did not reach significance or improve model fit, the authors concluded that their neuropsychological measures predicted SE outcomes equally well in patients with affective disorders and patients with psychotic disorders.

### Work-related outcomes

The most common work outcome was competitive job attainment during the follow-up period (*n* = 6) (46–48, 50, 51, 58), with one article (58) also including education aiming for competitive employment. Job attainment was defined as working in a competitive job at least for one day during the follow-up (47, 50, 51) or it was not further specified (46, 48, 58). Three (47, 50, 51) out of six articles found neuropsychological variables to be a significant predictor of job attainment. Regarding weeks worked (48, 52) and average weekly hours worked (49) over the study period, all three articles observed significant effects of neuropsychological variables, and regarding wages earned one (48) article found a significant effect of cognitive tests, while the other (52) did not.

### Follow-up period

The most common follow-up period was 2 years (*n* = 6), while 6 months (46) and 18 months (58) follow-up were only reported by one article each. To evaluate if the effect of cognitive predictors changed over time, McGurk et al. (49) added a time (quarters of 2-year period) by cognitive scores interaction into their predictive model, but the interaction was not significant and did not improve model fit.

### Control conditions

Among the eight included papers, only two study designs included a control group that did not receive any work-related intervention (treatment as usual; TAU) which were analyzed by two research articles included (50, 58). The interaction between study condition and cognitive variables was not significant in Metcalfe, Drake et al. (50). However, Christensen et al. (58) reported that cognitive functioning significantly predicted vocational recovery only in the IPS group compared with TAU and IPS plus cognitive and social skills training. The formal test of interaction effects, however, did not reveal a significant interaction as defined by the authors (*p* < 0.001). Landolt et al. (47) reported that the duration of SE (25, 40, or 55 h) did not influence the relationship between cognitive functioning and finding a job.

## Discussion

The aim of this scoping review was to identify and map research on the role of neuropsychological predictors of outcomes of vocational rehabilitation programs in people with major depressive disorder. The systematic literature search suggests that studies focusing exclusively on patients with major depression are missing when it comes to examining the impact of neuropsychological deficits on how individuals benefit from such programs. Moreover, research on samples with different diagnoses examining cognitive predictors that include patients with major depression is limited to only five study samples included with an estimated total of 1,103 (31%) depressive participants. In addition, we identified only four articles that focused on neuropsychology in their main research questions, and the included articles varied widely in terms of interventions, follow-up periods, diagnostic groups, outcome measures, neuropsychological tests, and covariates. However, SE and IPS, respectively, were the only rehabilitation approaches examined, suggesting that current research on cognitive predictors including persons with major depression mainly focuses on first place-then train (60) interventions. In sum, most articles found that neuropsychological measures significantly predicted work outcomes of SE interventions with global cognition scores, learning and memory tests, as well as processing speed and verbal fluency being relevant predictors. In several articles, neuropsychological variables were even significant when the important predictor work history was controlled for (46–49, 51), but due to the small number of studies and different study designs, more research targeting participants with major depressive disorder is needed to draw definite conclusions.

### Major depressive disorder diagnosis

The limited research focusing on depression is surprising, given that major depression is one of the most common mental disorders and among the leading causes for disability and early retirement (1). Many employees also experience negative effects of the depressive disorder on work participation and functioning, such as productivity loss or sickness absence. Although there is long-standing evidence of cognitive impairment and its association with psychosocial functioning in major depression (11, 16, 61), research on cognitive functioning in vocational rehabilitation programs that includes patients with major depression at all appears to have increased only recently, as all studies included in this review have been published only since 2016. This fits with the results of two reviews that could only include few studies on neuropsychological predictors of occupational functioning and the association of neuropsychology with employment (16, 17). In general, a recent review suggests that studies on vocational outcomes of IPS mainly include participants with schizophrenia and that existing studies might lack power to detect effects on people with major depression (62). To address diagnostic differences, IPS has recently been adapted for individuals with affective disorders to include “motivational, cognitive, and time use strategies” in addition to SE (63), but neuropsychological predictors have not yet been included.

However, diagnosis was not a significant predictor of work outcomes in any article and some authors concluded that their results apply independent of psychiatric diagnosis (48, 52). Only one article included the interaction between neuropsychology and diagnosis, but it supported the conclusion that neuropsychological measures are equally predictive of work outcomes of SE in persons with affective disorders as they are in persons with schizophrenia (49). This is noteworthy because it underlines how important neuropsychological aspects might be for returning to work also for patients with major depression, even if they showed less severe deficits in neuropsychological tests than persons with schizophrenia (48, 49, 52). Is has to be noted that several included studies did not distinguish between bipolar disorder and major depression, or major depression subtypes although they seem to show different patterns of cognitive impairment (64–67). In sum, research aiming at identifying diagnostic differences in neuropsychology predicting vocational outcomes of rehabilitation interventions is missing and more articles are needed to examine whether global cognition or certain domains predict outcomes differently for major depression and other diagnoses and in participants with different levels of impairment.

### Neuropsychological predictors of work outcomes

Because several studies aimed to identify the most relevant predictors of work outcomes, cognitive functioning was included in multivariate analysis alongside various other predictors of the success of the SE programs, like work history (46–51, 58), employment specialist competencies (46), or environmental factors (51). There was both evidence that cognitive functioning was a robust predictor even with these factors included and, on the other hand, that cognitive factors were no longer significant when other variables are included in the model (46, 58). The inconsistent results may be due to differences in measurement of predictors and outcomes, for instance the use of categorical or continuous measures of work history. It has been previously noted that a more precise coverage of important and complex constructs might contribute to a better understanding of IPS success (51).

The neuropsychological tests used covered a wide range of functional domains, but the instruments and the number of tests differed between articles. Given that some articles used the same samples, four out of five studies found a significant effect in their multivariate models predicting work outcomes of SE. Global test scores were significant predictors, as well as verbal learning and verbal and visual memory scores, which matches results of previous IPS studies on schizophrenia (68). The significance of global scores is in line with the finding that no specific pattern of cognitive deficits in depression has been identified yet and most studies show broad cognitive functions to be impaired (61). It also can be explained by the broad cognitive abilities that are required at the workplace and probably very different jobs that participants were searching. In general, modern jobs may more often require cognitive rather than physical abilities. Given that the actual impairment in cognitive functions differed between diagnoses and between studies, significant subdomains might reflect which cognitive abilities were especially relevant for participants’ jobs, such as verbal and visual abilities. However, the included studies on SE did not report which jobs their participants have had before, or which jobs they were aiming for. Including this in future research might allow to evaluate how cognitive functioning matches the cognitive requirements at the workplace, if it has an impact on vocational rehabilitation program outcomes, and how this is influenced by SE (29, 69).

The significant results regarding cognitive predictors of rehabilitation success suggest that even with individual vocational and psychosocial services, higher baseline cognitive functioning predicts better work-related outcomes. This is in line with studies showing that improvement in cognitive functioning can contribute to more positive outcomes of rehabilitation interventions (70, 71). Furthermore, cognitive remediation has the potential to improve work outcomes of rehabilitation interventions (38). Our results suggest that additional neuropsychological modules in such interventions may also be beneficial for people with depressive disorders. However, because only two articles included control groups without any intervention, the interaction between cognitive functioning and vocational interventions remains unclear. One model attempted to explain this interaction and the authors proposed that SE may compensate for some cognitive deficits (30). Our results support the suggestion that SE at least does not fully compensate for cognitive deficits in samples including patients with major depressive disorder. The question of whether specific subdomains may be compensated by SE and by what mechanisms is important for future research (47).

With regard to subdomains, it is noteworthy that executive functions did not significantly predict rehabilitation outcomes, although they have been associated with psychosocial functioning in depression before (72). As noted above, it is possible that SE compensated for deficits in executive functions (31), but according to the model by McGurk et al. (30) SE would compensate for basic cognitive functions such as attention rather than more complex functions. In addition, Porter et al. (66) noted several methodological issues to be considered when comparing significant with non-significant test results, such as the psychometric properties of the tests used (e.g., reliability or ceiling effects). Moreover, many tasks that are supposed to measure a certain cognitive domain often require many different cognitive functions (66). In the included articles, test scores that were used to measure a certain subdomain, such as processing speed (TMT-A; digit symbol coding test) or executive functioning (TMT-B), were used in other articles as a measure of global cognitive functioning, so in sum we cannot draw definite conclusions regarding most relevant subdomains.

Especially for individuals with major depressive disorder, future studies may consider including alternative definitions of cognitive impairment, because objective test results often do not match subjective impairment (73–76). Current studies suggest that the intraindividual differences between current deficits and premorbid cognitive functioning may be more relevant to subjective functional impairment at work than normative comparisons (77), because people are likely to have jobs that match their premorbid cognitive abilities and therefore are sensitive to deficits that would normatively be regarded unimpaired. Douglas et al. (64) showed that the amount of people defined as cognitively impaired increased significantly when correcting for premorbid IQ in inpatients with major depressive disorder. Individuals who have previously worked in an occupation with high cognitive demands may be more likely to subjectively perceive differences from their premorbid cognitive abilities and may not feel ready to return to work until they have not reached their premorbid levels, even if their test scores are normatively in the average range. In addition, even small cognitive deficits may still be relevant at the workplace for this group (17). Furthermore, performance on standardized tasks does not necessarily reflect real-life impairment in the workplace, because cognitive deficits increase in patients with major depression during experiences of failure (78) or emotionally negative distractions (79), which is more likely the case in the workplace. Moreover, there are several other illness related variables that are associated with cognitive impairment in major depression, such as rumination (80), attentional bias (81), and motivation (82, 83) which could be examined in terms of their interaction with neuropsychology and IPS outcomes.

Considering that the effect of cognition on employment status was only significant in half of the included articles that examined it, the outcome measure used by most SE studies, which is job attainment, may need to be broadened. A review suggests that cognitive deficits in major depressive disorder most importantly influence presentism rather than absenteeism (20) which has not been examined as an outcome variable in the included articles. Moreover, it has been suggested that cognitive functioning most importantly influences work behaviors or job tenure rather than work status (68, 84). If SE can support participants to return to work despite cognitive deficits, work productivity, job tenure, and the type of work also need to be considered especially in patients with major depression in order not to overlook functional impairment at the workplace. More diverse outcome variables would be even more relevant, when studies would include participants who already have a job and are on long-term sick leave due to their mental illness. All included studies targeted participants who were looking for a job, and interventions supporting participants returning to their job after a long sickness absence while considering cognitive functioning, appear to be missing.

### Study differences

Even though the included studies all applied SE and analyzed predictors of its success, there were substantial differences in terms of the interventions, follow-up periods, diagnostic groups, outcome measures, neuropsychological tests, and included covariates. This shows that there are a variety of applications and approaches (including a measure of cognitive functioning), but it also makes it difficult to compare results and draw conclusions from the small number of studies. Moreover, the samples came from four different countries, and the results need to be considered in terms of the labor market and social security services of each country. Another point to consider is that articles used different alpha levels to indicate significant results due to multiple testing or large sample sizes, which affects the interpretation of significant vs. non-significant results in this review.

### Limitations

Although we performed a systematic literature search using a wide range of different keywords and added a second, even broader search, our searching approach was limited to PubMed and PsychInfo and to the reference lists of included articles. Given the small number of resulting study samples, an additional search of other databases, such as Academic Search Premiere or Google Scholar, could have contributed to an even broader scope of this review. In addition, we did not search for gray literature. Another limitation concerns study selection, such that a few studies that fit the inclusion criteria did not further specify their sample’s diagnoses so that it was unclear if people with major depressive disorder were included at all (84–86). For instance, we decided not to include a study by Gold et al. (84), because the small subsample of participants with affective disorders was described as having “most often bipolar disorder.” Thus, the final number of participants with major depressive disorder remains unclear, and it could as well have been argued to include the study. Even in the included articles, precise information on the numbers of participants with depression was sometimes not available because affective disorders were grouped together (50, 51). Therefore, we could only estimate the total number of participants with major depressive disorder using data from the same baseline sample by McGurk et al. (49). Since the articles were based on samples with very different sample sizes and percentages of participants with major depressive disorder, no firm conclusions can be drawn from their results regarding the population of people with depression. A last point to keep in mind is that we decided to include several articles based on the same samples to reflect the full breadth of current research on our topic. In interpreting the study results, the findings of five articles drawn from only two samples should not be overestimated, even though one of these samples was particularly large.

## Conclusion

This scoping review identified eight articles (from five samples) that examined the role of objectively measured cognitive functioning in vocational rehabilitation intervention outcomes and included participants with major depressive disorder. The included articles examined cognitive predictors in the context of recent IPS or SE trials, respectively, in North America and Europe with varying add-on interventions, research questions, outcomes, and covariates. Although major depression in particular or diagnostic differences were not focused, the articles suggest that higher cognitive functioning predicts better work related outcomes, similar to findings in schizophrenia. Thus, additional neuropsychological modules during vocational programs might further improve work-related outcomes for people with depression. However, as most studies did not include control participants who did not receive an intervention, the interplay between cognitive performance and the vocational rehabilitation intervention regarding work success remains unclear. Future research may also consider to include depression related covariates and broader outcomes, and examine their interactions with neuropsychological measures. Evidence that global measures of cognition, as well as certain subdomains have a significant impact on certain vocational outcomes also requires further investigation in individuals with major depressive disorder. More focus could be given to individual workplaces and their interactions with differently defined subjective and objective cognitive deficits and varying rehabilitation outcomes.

## Data availability statement

The original contributions presented in this study are included in the article/supplementary material, further inquiries can be directed to the corresponding author.

## Author contributions

JB and LD conceptualized the review, searched and screened articles, and extracted data from the included articles. PS was involved in the literature search and screening process. JB prepared the initial draft of the manuscript, which was revised by LD. MD and TB participated in the interpretation of the results and critically reviewed the manuscript draft. All authors read and approved the final version of the manuscript.

## Footnotes

1
https://osf.io/5yrnf
